# Epidemiology of hospital-acquired bloodstream infections in Belgium: a retrospective dynamic cohort study, 2000-2014

**DOI:** 10.1186/2049-3258-73-S1-P27

**Published:** 2015-09-17

**Authors:** Koen Blot, Naïma Hammami, Stijn Blot, Dirk Vogelaers, Marie-Laurence Lambert

**Affiliations:** 1 Universiteit Gent, Gent, Belgium; 2 Scientific Institute of Public Health, Brussels, Belgium; 3 Ghent University Hospital, Ghent, Belgium

## Introduction

Hospital-acquired bloodstream infections (HABSI) cause increased morbidity, mortality, and hospital costs that are partially preventable. The Belgian *Surveillance of Septicaemia in Hospitals* programme allows optional HABSI surveillance analysis.

## Methods

The study population was a dynamic cohort of 110 Belgian hospitals (60.032 HABSI) participating at least five years during 2000-2014. Mixed-effects negative binomial regression analysis calculated the adjusted incidence rate ratios (IRR) with 95% confidence interval (CI) for annual trends and seasonal variation of HABSI rates per 10.000 patient-days. Antibiotic-resistant phenotype analysis was performed for 2013-2014, when reporting became mandatory.

## Results

Regression analysis identified a decreasing total HABSI trend (IRR -0.5% [-0.8; -0.1%]; p=0,006) with increases in 2013-2014. There were decreasing annual rates of coagulase-negative staphylococci ([CoNS] IRR -4.4%; p<0.001), *S. aureus* (IRR -0.8%; p=0.016), and *Candida* (IRR -1.3%; p=0.004); and increases in *E. coli* (IRR +2.8%; p<0.001), *Klebsiella* (IRR +2.8%; p<0.001), and *Enterococcus* species (IRR -2.6%; p<0.001). *Pseudomonas spp.* incidence decreased among intensive care units (IRR -3.0%; p<0.001). Seasonality was present and pronounced with summer peaks amongst *Acinetobacter* (IRR +80%; p=0.002), *Klebsiella* (IRR +69%; p<0.001), *Enterobacter* (IRR +67%; p<0.001), and *Pseudomonas* species (IRR +28%; p<0.001); and winter spikes for *Streptococcus spp.* (IRR +32%; p<0.001). HABSI of unknown origin rates declined (IRR -5.7%; p<0.001). Rates of carbapenem-resistant *Pseudomonas* increased during the 2^nd^ trimester (IRR +50%; p=0.03).

## Conclusion

The results identified a declining total HABSI rate trend with increases during the last three years. There are increasing proportions of Gram-negative pathogens, which exhibited surges during the summer periods. Strengths of this study include the long-term span including multiple nationwide hospitals. Limitations involve lack of patient case-mix and risk-adjustment. Decreases of HABSI of unknown origin and CoNS may represent improved diagnosis and contamination identification. Considering the prominent seasonal variation, the relevance of regular colonisation cultures in hospitalised patients remains to be investigated.

